# Development and Evaluation of an Orodispersible Tablet Formation for the Delivery of a Hydrophobic Drug

**DOI:** 10.1155/2024/7914860

**Published:** 2024-10-17

**Authors:** Razan Haddad, Ahmed R. Gardouh

**Affiliations:** ^1^Department of Pharmaceutical Sciences, Faculty of Pharmacy, Jadara University, Irbid 21110, Jordan; ^2^Department of Pharmaceutics and Industrial Pharmacy, Faculty of Pharmacy, Suez Canal University, Ismailia 41522, Egypt

## Abstract

Orodispersible tablet (ODT) is a promising avenue for drug delivery, offering a dosage form that can be disintegrated instantaneously in the mouth and released the drug that dissolves or disperses in the saliva without the addition of water. ODT can effectively boost the dissolution rate and consequently the bioavailability of several hydrophobic drugs. Additionally, ODT is very attractive and suitable for specific patients who are unable to swallow the traditional tablet. The basic approach in the fabrication of oral tablets for hydrophobic drugs relies on the utilization of superdisintegrants which allow prompt disintegration of tablets after swallowing. In the present investigation, escitalopram oxalate was chosen as a model drug, which is a hydrophobic, antidepressant, selective serotonin reuptake inhibitor (SSRI) drug. Nine formulas of escitalopram oxalate ODTs were prepared by varying the concentrations of three different superdisintegrants: sodium starch glycolate, croscarmellose sodium, and crospovidone to improve the dissolution and release of escitalopram oxalate. Each was used in three different concentrations (2.5%, 5%, and 7.5%), and all the ODTs were prepared by the direct compression method. The micrometric characterization of the powder blend used in the formulations was investigated such as angle of repose, bulk and tapped densities, compressibility percent (Carr's index), and Hausner ratio. Furthermore, the prepared ODTs were characterized in terms of weight variation, thickness, diameter, hardness, friability, in vitro disintegration, wetting time, water absorption ratio, drug content, in vitro dissolution, and accelerated stability study. The results showed that the formula (ODT9) that contained 7.5% of the superdisintegrant sodium starch glycolate had superior characteristics in almost all the tests, with a dissolution rate of 100% after 6 minutes. Also, it was stable under the accelerated stability conditions.

## 1. Introduction

Oral dosage forms are considered the most favorable and convenient route of drug administration [1, 2]. The orodispersible tablet (ODT) is a delivery system that promptly disintegrates in the mouth once it contacts the saliva; hence, there is no need for water during ingestion. ODT stands as a prominent and innovative dosage form and it has garnered considerable attention due to its unique characteristics, offering a convenient and patient-friendly approach to drug administration. However, ODT has other known names such as orally disintegrating tablets (ODT), quick/rapid melt tablets, fast/rapid dissolving or disintegrating tablets (FDTs), mouth-dissolving/disintegrating tablets (MDTs), and porous tablets [3, 4].

ODTs have several pros over conventional tablets such as rapid fragmentation, quick onset of action, and improved patient compliance, particularly for pediatric, geriatric, psychiatric, paralyzed, and bedridden patients. As the ODT is absorbed directly after administration through the pregastric mucosa within seconds, swallowing difficulty (dysphasia) problems are ignored [5–7]. Furthermore, the first-pass metabolism effect can be avoided in the ODTs, which enhances the bioavailability and subsequently reduces the adverse effects and the frequency of dosing [8]. Also, many studies indicated that ODT is cost-effective and more efficient than the traditional tablet dosage form [8–10]. However, the development of ODT demands a comprehensive understanding of several features, which include manufacturing methods, compatibility with different APIs, taste-masking approaches, stability issues, and regulatory considerations [11, 12].

Various methods and techniques have been used to prepare ODTs including the lyophilization (freeze-drying) method, the molding method (compression modeling or cotton candy process), and the compaction method (direct compression method). However, the compaction method is considered the most advantageous and common method due to several features such as cost-effectiveness, established manufacturing technique, ease of process and performance on large-scale production, and suitability for high drug doses [7, 13].

Superdisintegrants are mainly used to enhance the efficiency of the ODT by aiding in the disintegration or breaking of the tablet into small fragments that can be dissolved quickly and easily. They are generally used at low concentrations (1%–10%) based on the weight of the dosage form [14, 15]. Primojel (sodium starch glycolate), Polyplasdone XL (crospovidone), and Ac-Di-Sol (croscarmellose sodium) are often preferred over other superdisintegrants due to their superior performance and efficiency in specific formulations of fast-disintegrating dosage forms like ODTs. For instance, these superdisintegrants have several favorable properties such as fast disintegration time, efficiency at low concentrations, nongelling properties, compatibility with other drugs and excipients, and robust performance at variable pH ranges [14, 16].

Escitalopram oxalate is an orally administered selective serotonin reuptake inhibitor (SSRI) that is mainly used to treat major depressive disorder (MDD) and several anxiety disorders [17]. It is a hydrophobic drug belonging to the BCS class II which contains drugs that have low solubility and high permeability [18]. Escitalopram oxalate is available in the market under various trade names, such as Lexapro ODT, Nexito-MD, S-Citadep ODT, and Generic Escitalopram ODT. Each formula has its pros and cons. However, the aim is to develop an ODT formula that is easily manufactured, stable, and affordable for patients [14, 19].

In this study, escitalopram oxalate was used as a model drug due to its widespread use in treating psychiatric disorders, which often necessitate long-term and consistent medication adherence as these conditions are usually accompanied by difficulties in swallowing, particularly in elderly patients or those with cognitive impairments, making ODTs a suitable delivery system. Furthermore, the hydrophobic nature of escitalopram oxalate presents formulation challenges, particularly in ODTs, making it an ideal candidate for investigating strategies to enhance the solubility and bioavailability of such drugs. By focusing on escitalopram oxalate, this study aims to provide a formulation approach that could be extrapolated to other hydrophobic drugs, thereby contributing to the broader field of pharmaceutical formulation science. Escitalopram oxalate ODT was prepared using the direct compression method as it is considered a simple, cost-effective, and direct technique in pharmaceutical manufacturing. Briefly, various excipients (superdisintegrants) and concentrations were utilized to fabricate nine different formulations of ODTs. Then, they were characterized and compared to end up with the most optimum and efficient formula.

## 2. Materials and Methods

### 2.1. Chemicals and Reagents

Escitalopram oxalate was kindly donated by Shiba Pharma Co. Croscarmellose sodium (Ac-Di-Sol), sodium starch glycolate (Primojel), and microcrystalline cellulose (Avicel PH102) were purchased from Blanver, Brazil. Mannitol and sodium hydroxide were purchased from Oxford Lab, Mumbai (India). Crospovidone (Polyplasdone XL) was purchased from ISP, Switzerland. Methanol and sodium dihydrogen phosphate were purchased from PureLab, Madison (USA).

### 2.2. Construction of Standard Calibration Curve of Escitalopram Oxalate at 239 nm

The stock solution of escitalopram oxalate (15 *μ*g/mL) was prepared. Concentrations of 2, 4, 6, 8, and 10 *μ*g/mL of escitalopram oxalate were prepared by taking (2, 4, 6, 8, and 10 mL, respectively) of the stock solution and completed to 10 mL with blank. The different drug concentrations were measured spectrophotometrically using a UV spectrophotometer (Hitachi, U-2900 UV spectrophotometer, Japan) at the maximum wavelength calculated (239 nm) using a mixture of 1 M NaOH and methanol as blank. The absorbance values were plotted against the corresponding concentrations to obtain the linear calibration [20].

### 2.3. Construction of Standard Calibration Curve of Escitalopram Oxalate in Phosphate Buffer pH 7.4

The stock solution of escitalopram oxalate (10 *μ*g/mL) was prepared. Concentrations of 1, 2, 3, 4, 5, 6, 7, 8, 9, and 10 *μ*g/mL of escitalopram oxalate were prepared by taking (1, 2, 3, 4, 5, 6, 7, 8, 9, and 10 mL, respectively) of the stock solution and completed to 10 mL with blank. The different drug concentrations were measured spectrophotometrically using a UV spectrophotometer (Hitachi, U-2900 UV spectrophotometer, Japan) at the maximum wavelength calculated (237 nm) using phosphate buffer pH 7.4 as blank. The absorbance values were plotted against the corresponding concentrations to obtain the linear calibration [20].

### 2.4. Preparation of Escitalopram Oxalate ODTs

**Figure 1:**
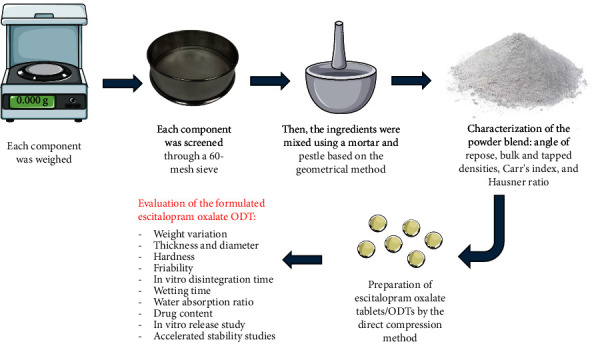
A flowchart illustrating the preparation process of the ODTs⁣^∗^. ⁣^∗^This figure was performed using the SMART Servier Medical Art website https://smart.servier.com/.

Nine formulas of escitalopram oxalate ODTs were prepared by varying the concentrations of three different superdisintegrants: croscarmellose sodium (Ac-Di-Sol), sodium starch glycolate (Primojel), and crospovidone (Polyplasdone XL) using three diverse concentrations of each superdisintegrants (2.5%, 5%, and 7.5%) [15].  Table 1 represents the detailed components of all formulations. Escitalopram oxalate ODTs were fabricated using the direct compression approach. Briefly, each component (excipient) was screened through a 60-mesh sieve (Sieve No. 60, USA standard test sieve, ASTME-11 specification, Gilson Company, USA) individually and then mixed with a mortar and pestle. The active agent and microcrystalline cellulose were combined gradually in small amounts to obtain a homogenous blend at each time. After that, all the remaining constituents were weighed and mixed based on the geometrical manner except for magnesium stearate, which was added at the end of the mixing process. At this stage, the powder blends were characterized by performing the precompression analysis of the powder blend. Finally, the tablets were compacted employing an 8 mm flat-faced punch to yield tablets weighing 120 mg each, utilizing a 10-station rotary tablet compression machine (pilot press tablet compression machine; model CPMD-10 manufactured by Chamunda Pharma Machinery Private Limited, India). At this stage, the prepared tablets were characterized by the evaluation of postcompression parameters [21].  Figure 1 illustrates the preparation process of the ODTs.

Regarding formulation of escitalopram oxalate tablets, a combination of microcrystalline cellulose (Avicel PH102) and mannitol was chosen as a diluent as the former is commonly used as a filler and binder, especially in the direct compression method due to its good flowability and compressibility. Also, mannitol is useful in ODT formulations due to its sweet taste, cooling effect, and optimum mouthfeel. Furthermore, as a fast disintegration is needed in the ODT formulation, superdisintegrants like Primojel (sodium starch glycolate), Polyplasdone XL (crospovidone), and Ac-Di-Sol (croscarmellose sodium) were used. Finally, the elegant shape of the tablet can be obtained by the commonly used lubricants such as magnesium stearate and talc [22, 23].

### 2.5. Precompression Analysis of Powder Blend

The following parameters were determined to evaluate the powder blend, and Table 2 illustrates the acceptable range for these parameters [24–27].

#### 2.5.1. Angle of Repose

The measurement of the angle of repose involved passing the powder blend through a funnel consistently positioned at the same altitude during all experiments. The parameters used were the height (*h*) and radius (*r*) of the formed cone. The angle of repose (*θ*) was determined using the following equation [28]:

(1) $$\begin{array}{c} \text{Tan}\theta =\frac{h}{r}. \end{array}$$

#### 2.5.2. Bulk and Tapped Densities

Measurement of both bulk density (Db) and tapped density (Dt) was obtained. In brief, a sample of 5 g of a powder blend was introduced into a 10-mL measuring cylinder, and the volume of this blend was recorded first before any tapping. Afterward, the cylinder underwent tapping until no additional alterations in volume were observed. The determination of Db and Dt was carried out utilizing the subsequent equations [28]:

(2) $$\begin{array}{c} \text{Db}=\frac{\text{weight}}{\text{bulk volume}\left(\text{Vb}\right)},\\ \text{Dt}=\frac{\text{weight}}{\text{tapped volume}\left(\text{Vt}\right)}. \end{array}$$

#### 2.5.3. Carr's Index

Carr's index is also called compressibility percent. As the compressibility of the powders can be correlated indirectly to various factors such as relative flow rate, cohesiveness, and particle size, the compressibility percentage can be measured indirectly using the tap and bulk densities based on the subsequent equation [28]:

(3) $$\begin{array}{c} \text{compressibility }\%=\left(\frac{\text{Dt}-\text{Db}}{\text{Dt}}\right)\times 100\%. \end{array}$$

#### 2.5.4. Hausner Ratio

The relationship between the bulk density and tapped density can be utilized to obtain Hausner ratio which provides a valuable assessment of the flow characteristics of powder particles [28].

(4) $$\begin{array}{c} \text{Hausner ratio}=\frac{\text{Dt}}{\text{Db}}. \end{array}$$

### 2.6. Postcompression Evaluation of the Formulated Escitalopram Oxalate ODT

#### 2.6.1. Weight Variation

Twenty escitalopram oxalate ODTs for each formula were chosen accidentally, and the average weight was obtained using an electrical balance (SARTORIUS, TE2145, Germany). After that, individual tablets were weighed and evaluated in comparison with the average weight [7, 21]. The mean ± standard deviation (SD) was calculated.

#### 2.6.2. Thickness and Diameter

The thickness and diameter of escitalopram oxalate ODTs were determined employing vernier caliber (model KH-97105-00, USA). They are expressed in millimeters [7, 21]. Ten tablets were used to perform the test, and the mean ± SD was calculated.

#### 2.6.3. Hardness

The hardness of a tablet, also known as tablet crushing strength (Fc), denotes the force necessary to fracture a tablet through diametric compression. This parameter was assessed employing a Campbell tablet hardness tester (model-C-DHT 100 manufactured by Campbell Electronics, India) [21]. It is represented in kg/cm^2^. The hardness test was performed on 10 tablets, and the mean ± SD was calculated.

#### 2.6.4. Friability

The evaluation of friability in tablets containing escitalopram oxalate was conducted utilizing the Roche Friabilator (model EF2 manufactured by Electrolab, India). This apparatus exposes the tablets to a dual influence of abrasion and shock within a plastic chamber, which rotates at 25 rpm, and the tablets were overthrown from an elevation of 6 inches during each rotation. A sample of the preweighed tablets was introduced into the friabilator, and the tablets underwent 100 rotations. Subsequently, the tablets were gently cleaned and dusted with a soft muslin cloth, and their weights were reassessed [7, 21]. The friability assessment involved 20 tablets, and the mean ± SD was computed. The percentage friability (F) is determined using the subsequent formula:

(5) $$\begin{array}{c} F=\left(\frac{W_{\text{initial}}-W_{\text{final}}}{W_{\text{initial}}}\right)*100, \end{array}$$

where *W*_initial_ refers to the initial weight of the tablets and *W*_final_ refers to the weight of the tablets after the friability test.

#### 2.6.5. In Vitro Disintegration Time

Escitalopram oxalate tablets were introduced into 10 mL of phosphate buffer solution with a pH of 1.2, representing gastric pH, at a controlled temperature of 37 ± 0.5°C in a disintegration tester (Model DIST-3, D-63512 Hainburg by Pharma Test, Germany 2019). The duration necessary for the tablets to disintegrate was recorded [7, 13, 22]. This experiment was performed on 10 tablets, and the mean ± SD was subsequently computed.

#### 2.6.6. Wetting Time

The wetting time of escitalopram oxalate tablets was determined through a straightforward technique. Briefly, five circular Whatman filter papers (no. 1) were evenly arranged in a Petri dish, covering its entire area. Then, 10 mL of acidic buffer solution has a pH of 1.2 was placed in the Petri dish, at a temperature of 37 ± 0.5°C. A tablet was precisely positioned on each filter paper, and the duration for water to dampen the upper surface of the tablets was recorded as the wetting time [7, 21]. This procedure was operated on five tablets, and the mean ± SD was computed.

#### 2.6.7. Water Absorption Ratio

The weight of escitalopram oxalate tablets before placing them in the Petri dish was recorded as wb. Furthermore, the weight of the tablet after wetting was obtained (wa) carefully using a balance. Based on that, the water absorption ratio was obtained utilizing the subsequent equation [7, 21]:

(6) $$\begin{array}{c} R=100\times \frac{\left(wa-wb\right)}{wb}, \end{array}$$

where *wa* corresponds to the weight of the wet tablet and *wb* refers to the initial weight of the tablet. This experiment was conducted on five tablets, and the mean ± SD was obtained.

#### 2.6.8. Drug Content

Twenty tablets containing escitalopram oxalate were meticulously weighed and then pulverized. An aliquot of the resulting powder, corresponding to 30 mg of escitalopram oxalate, was meticulously transferred to a dry, clean, and calibrated 100-mL volumetric flask. Subsequently, it was dissolved in 10 mL of 1 M NaOH, then 40 mL of methanol was added, and the mixture was thoroughly mixed for 5 min. An additional 40 mL of methanol was introduced, and the mixture was subjected to magnetic stirring for 3 h. After cooling, the flask received an ample amount of methanol to achieve a total volume of 100 mL and underwent filtration, resulting in the creation of a stock solution with a concentration of 300 *μ*g/mL. A 5 mL aliquot was withdrawn from the stock solution and augmented to 100 mL with methanol, yielding a solution with a concentration of 15 *μ*g/mL [7, 15, 21]. This solution underwent drug content analysis at 239 nm utilizing a UV spectrophotometer (Hitachi, U-2900 UV spectrophotometer, Japan). The entire procedure was executed in triplicate, and the mean ± SD was subsequently calculated.

#### 2.6.9. In Vitro Release Study

The dissolution behavior of escitalopram oxalate in various formulations was investigated through in vitro studies employing a US Pharmacopeia (USP) dissolution test apparatus Type II (paddle) (USP Dissolution Tester, six-cup model, Apparatus II, Erwika. Apparatebau GmbH, Germany) operating at 50 rpm. A 900 mL of phosphate buffer with a pH of 7.4 was employed as a dissolution media, and the temperature was meticulously set at 37 ± 0.5°C. At specified time intervals (1, 2, 4, 6, 8, 10, 12, 15, and 20 min), a 5 mL aliquot was withdrawn, filtered via Whatman filter paper, and spectrophotometrically analyzed at 362 nm using a UV-Visible spectrophotometer [29]. To ensure a constant volume throughout the test, an equal volume of prewarmed fresh medium at 37°C was promptly replaced after each sampling [7, 21, 30]. The entire study was conducted in triplicate.

#### 2.6.10. Kinetic Treatments and Parameters for the In Vitro Release of Escitalopram Oxalate ODTs

The acquired data from the in vitro release study of escitalopram oxalate ODTs underwent analysis using different kinetic models to elucidate the release mechanisms for each formulated variant. Specifically, the formulated tablets were subjected to zero-order, first-order, and Higuchi's diffusion model assessments. For each formula of the nine prepared formulations, the correlation coefficients and kinetics parameters were estimated concerning the in vitro release profiles of escitalopram oxalate. For each model, the coefficient of determination (*R*^2^) was determined. The drug release kinetics model exhibiting the highest value of the coefficient of determination, closely approaching 1, was identified as the most suitable and best-fitted model [31].

#### 2.6.11. Accelerated Stability Studies of Escitalopram Oxalate Tablets

Stability studies were performed on all the prepared escitalopram oxalate ODT formulations by keeping the samples in electrical hot air ovens at (40°C) and (60°C) with a relative humidity set at 75%, achieved through the use of a saturated sodium chloride solution. These studies were performed for 6 months. During each time point (1 month), samples were withdrawn and subjected to testing for hardness, in vitro drug release, in vitro disintegration time, and drug content. The acquired data from the stability study of escitalopram oxalate tablets underwent analysis utilizing different kinetic orders to elucidate the degradation mechanisms for each formulation. Specifically, the formulated escitalopram oxalate tablets were evaluated using zero-, first-, and second-order kinetics. The kinetic investigations involved the calculation of kinetic parameters and correlation coefficients, providing insights into the shelf stability of escitalopram oxalate [31].

The determination of the best kinetic order for the degradation of escitalopram oxalate formulations was based on the largest values of the obtained correlation coefficients. Through this experiment, specific reaction rate constants based on the elevated temperatures were determined employing the Arrhenius equation. This involved substituting the specific rate constants established through the investigation at the two raised temperatures [32].

The activation energy was computed operating this equation:

(7) $$\begin{array}{c} \mathrm{Log}\left(\frac{K_{2}}{K_{1}}\right)=\left(\frac{\text{Ea}}{2.303R}\right)\times \left[\frac{\left(T_{2}-T_{1}\right)}{\left(T_{2}T_{1}\right)}\right], \end{array}$$

where *K*_1_ is the specific reaction rate constant at temperature *t*_1_, *K*_2_ is the specific reaction rate constant at temperature *t*_2_, Ea is the energy of activation, *R* is the gas constant (1.987 Cal./degree mole), *T*_1_ is the absolute temperature for *t*_1_, and *T*_2_ is the absolute temperature for *t*_2_.

Based on that, the decomposition reaction rate constant was anticipated at 25°C (room temperature), *K*_2_, and by a second substitution in the Arrhenius equation utilizing the determined activation energy and one of the rate constants obtained at the elevated temperatures [32].

Once *K*_2_ was determined, it enabled the calculation of the half-life and the time required for the tablets to lose 10% of their drug content. Furthermore, *t*_90_ represents the duration during which the tablets stay in compliance with the official conditions for drug content. The ability to calculate these parameters provides valuable insights into the stability and shelf-life of the escitalopram oxalate formulations under real-world storage conditions, aiding in ensuring the quality and efficacy of the pharmaceutical product over time [32].

## 3. Results and Discussion

### 3.1. Analysis of Escitalopram Oxalate

#### 3.1.1. Construction of Standard Calibration Curve of Escitalopram Oxalate in Methanol at 239 nm

A calibration curve for escitalopram oxalate was generated using the UV spectrophotometer by measuring the absorbance at a wavelength of 239 nm in methanol. It was determined that the calibration curve for escitalopram oxalate adhered to Beer's Lambert law within the concentration extent from 0 to 10 *μ*g/mL. The constants obtained were 0.034 for the slope, 0.03 for the intercept, and 0.9999 for r2 (*y* = 0.034*x* − 0.03).

#### 3.1.2. Construction of Standard Calibration Curve of Escitalopram Oxalate in Phosphate Buffer pH 7.4 at 237 nm

A calibration curve for escitalopram oxalate was generated using the UV spectrophotometer by measuring the absorbance at a wavelength of 237 nm in phosphate buffer (pH 7.4). It was determined that the calibration curve for escitalopram oxalate adhered to Beer's Lambert law within the concentration extent from 0 to 10 *μ*g/mL [30]. The constants obtained were 0.033 for the slope, 0.111 for the intercept, and 0.9994 for r2 (*y* = 0.033*x* + 0.106).

### 3.2. Precompression Analysis of Powder Blend

Precompression analysis of powder blend is the investigation of micrometric properties of prepared escitalopram oxalate powder blends, which includes the angle of repose, determination of the bulk and tapped densities, Hausner ratio, and compressibility percent.

#### 3.2.1. Angle of Repose

It is a direct method for estimating escitalopram oxalate flowability. The recorded values for the angle of repose of the escitalopram oxalate of the powder mixture varied between 31.96° and 38.75°, as represented in Table 3. The outcomes signify that the formulations of escitalopram oxalate exhibit commendable to satisfactory flowability. Noteworthy is the observation of superior flowability in four formulations (ODT9, ODT8, ODT4, and ODT1), while five formulations demonstrated a fair level of flowability (ODT2, ODT7, ODT5, ODT6, and ODT3). Consequently, it is deduced that the meticulously prepared mixture of escitalopram oxalate powder exhibits free-flowing characteristics, rendering it suitable for direct compression [33].

This ranking infers that the inclusion of sodium starch glycolate in the preparation of escitalopram oxalate ODT significantly enhances flowability compared to other employed superdisintegrants, namely, crospovidone and croscarmellose sodium. Escitalopram oxalate, inherently possessing poor flowability, demonstrates an incremental improvement in flowability corresponding to increasing concentrations of sodium starch glycolate, with ODT9 (7.5% sodium starch glycolate) exhibiting higher flowability in comparison with ODT8 (5% sodium starch glycolate) and ODT7 (2.5% sodium starch glycolate). This finding underscores the pivotal role of sodium starch glycolate in optimizing the flow characteristics of the escitalopram oxalate powder blend, highlighting its pronounced advantage over the other superdisintegrants (crospovidone and croscarmellose sodium). Escitalopram oxalate itself has very poor flowability; the higher the sodium starch glycolate concentrations, the better flowability obtained or calculated, i.e., the angle of repose for ODT9 (7.5% sodium starch glycolate) > ODT8 (5% sodium starch glycolate) > ODT7 (2.5% sodium starch glycolate).

#### 3.2.2. The Bulk and Tapped Densities

The flow characteristics of the formulated escitalopram oxalate powder mixture were evaluated indirectly through the determination of both bulk and tapped densities. Those parameters, besides the derived Hausner ratio and Carr's index, serve as crucial indicators of the powder's flow characteristics. The bulk and tapped densities, as well as the corresponding equations for their determination, are shown in Table 3, providing essential perceptions of the flow properties of the formulated mixture of powders. The Db measurements of the escitalopram oxalate powder mixture were observed to range from 0.417 gm/cm^3^ (noted in ODT4 and ODT7) to 0.500 gm/cm^3^ (observed in ODT3). Simultaneously, the tapped densities exhibited a range from 0.556 gm/cm^3^ (in ODT9) to 0.625 gm/cm^3^ (in ODT3).

#### 3.2.3. The Hausner Ratio

The Hausner ratio, a key metric reflective of powder or granular material flowability, was estimated based on the bulk and tapped densities. The obtained Hausner ratio values varied from 1.178 (in ODT9) to 1.364 (in ODT4), as detailed in Table 2. A Hausner ratio less than 1.25 is indicative of superior flowability, and accordingly, the results demonstrate that five formulations exhibit good flowability (ODT9, ODT2, ODT1, ODT8, and ODT3), while the remaining four formulations display acceptable flowability (ODT5, ODT6, ODT7, and ODT4) [34].

This hierarchical arrangement provides valuable insights into the comparative flow properties of the different formulations, emphasizing the role of specific ingredients in influencing the powder blend's flow characteristics.

#### 3.2.4. Compressibility % (Carr's Index)

The compressibility percent, serving as an indirect indicator of relative flow rate, plays a pivotal role in evaluating the flow properties of the escitalopram oxalate powder blend. Compressible materials tend to exhibit reduced flowability. The obtained compressibility percent values fall into distinct ranges, each associated with different levels of flowability. Values between 5 and 12 indicate excellent flowability, 12 to 16 suggest good flowability, 18 to 21 signify fair passable flowability, 23 to 35 denote poor flowability, and 33 to 38 indicate very poor flowability [29]. Remarkably, the ranking order derived from compressibility percent data aligns with that taken from the data of the Hausner ratio [34].

Carr's index, a further measure of powder flow properties, reveals the best value as 15.094 for ODT9 and the least favorable as 26.667 for ODT4, as detailed in Table 2.

The conclusion drawn is that formulations holding sodium starch glycolate demonstrate the best flowability, followed by those containing croscarmellose sodium and crospovidone. Moreover, an intriguing observation is the positive correlation between the strength of sodium starch glycolate (ranging from 2.5% to 7.5%) and the enhanced flow characteristics of the powder mixture. The ninth formula, which incorporated 7.5% sodium starch glycolate, emerges as the leading formulation due to its superior flow properties concerning the other prepared formulations.

### 3.3. Preparation of Escitalopram Oxalate Tablets by Direct Compression Method

Escitalopram oxalate was formulated as tablets employing the direct compression technique. In this investigation, three superdisintegrants were employed, i.e., sodium starch glycolate (Primojel), croscarmellose sodium (Ac-Di-Sol), and crospovidone (Polyplasdone XL) using various concentrations (2.5%, 5%, and 7.5%). The formulation process resulted in the creation of nine different formulas of escitalopram oxalate tablets, each characterized by a specific combination of superdisintegrant type and concentration. The details of the suggested formulas, encompassing the type and concentration of superdisintegrants used, are comprehensively presented in Table 1, offering a comprehensive overview of the experimental design employed in this study.

### 3.4. Evaluation of the Formulated Escitalopram Oxalate Tablets

The assessment of all batches of escitalopram oxalate tablets encompassed a thorough examination of diverse postcompression parameters, including but not limited to weight variation, thickness, diameter, hardness, friability, in vitro disintegration time, drug content, wetting time, and water absorption ratio. The detailed findings about these postcompression parameters for each formulation are meticulously documented in Table 4, providing a comprehensive overview of the tablets' attributes.

#### 3.4.1. Weight Variation

The calculated weights of the escitalopram oxalate tablets fell within the range of 199.6–202.1 mg, as delineated in Table 4. The observed weight variation was within the acceptable limits specified in pharmacopeias (±7.5%). According to the USP standards, deviations for tablets depend on their weight categories: For tablets < 80 mg, the permissible deviation is ±10%; for tablets ranging between 80 and 250 mg, the acceptable deviation is ±7.5%; and for tablets > 250 mg, the deviation should not exceed ±5%. It is noteworthy that all formulations of escitalopram oxalate tablets successfully passed the weight variation test, demonstrating uniform weights with low SD values [35].

#### 3.4.2. Thickness and Diameter

The thickness of all the prepared escitalopram oxalate tablets varied from 3.808 to 3.877 mm which reflects a uniformity of the tablets as demonstrated in Table 4. The diameter of all tablets from the nine formulas was between 8.071 and 8.08 mm. Ten tablets of each formula were assessed for the thickness test and the outcomes aligned with the specifications outlined in the USP, where *a* ± 5% limit is deemed acceptable, contingent upon the size of the tablet [35].

#### 3.4.3. Friability Test

The friability test results for the escitalopram tablets were within the stipulated bounds, with the friability percentages for all formulations being less than 1%, as listed in the USP [7]. The recorded loss in total weight due to friability ranged from 0.3718% (ODT9) to 0.7894% (ODT1), as detailed in Table 4. Markedly, none of the formulations exceeded a friability value of 0.79%. These results indicated that the escitalopram oxalate tablets exhibited mechanical stability, demonstrating their resistance to the stresses of transportation and handling [35].

The maximum friability value (0.79%) was recorded by ODT1, while the minimum one (0.37%) was reported by ODT9.

#### 3.4.4. In Vitro Disintegration Time

The in vitro disintegration time results for the prepared escitalopram oxalate tablets fell within the prescribed limits, adhering to the criteria for ODTs. The recorded values ranged from 8.3 to 21.9 s, as represented in Table 4. Remarkably, formulations consisting of sodium starch glycolate exhibited rapid disintegration, then those with crospovidone and finally croscarmellose sodium [35].

Escitalopram oxalate tablets varied in the in vitro disintegration time values from 8.3 s for ODT9 to 21.9 s for ODT1. Additionally, it was noted that the increment of the superdisintegrant concentration from 2.5% to 7.5% resulted in a reduction in disintegration time. However, an exception to this trend was noted in formulations containing crospovidone, where an increase beyond 5% concentration led to an elongation of disintegration time. The effectiveness of superdisintegrants, such as sodium starch glycolate and crospovidone, relies on their ability to swell significantly when exposed to water, creating channels for water-wicking and subsequent swelling. For sodium starch glycolate, concentrations between 5% and 10% are optimal, ensuring sufficient channels for wicking and swelling without compromising tablet hardness. Crospovidone, with its remarkedly porous structure, efficiently attracts significant quantities of water through a water-wicking mechanism, and its effective concentration range is between 2% and 5%. Beyond 5%, there is a risk of increased disintegration times due to gelling and viscosity effects [36].

#### 3.4.5. Hardness

The hardness of tablets provides significant indications regarding tablet withstanding handling storage and shipping processes, and to be acceptable, it should be between 2 and 8 kg/cm^2^ [30]. According to the results, the hardness values listed range from 4.21 (ODT5) to 4.55 (ODT6 and ODT7) as all the values for the ODT formulations are acceptable. However, the variance in these values is mainly affected by changing the type and concentration of the super disintegrant and as mentioned in the previous paragraph (Section 3.4.4). For instance, if the formulation contains an optimal level of an effective superdisintegrant such as sodium starch glycolate (Primojel), croscarmellose sodium (Ac-Di-Sol), or crospovidone, it can facilitate rapid tablet disintegration even in the presence of high hardness. Sodium starch glycolate, for example, in ODT9, has shown rapid swelling and hydration, which showed faster disintegration, even when the tablet is densely compacted [30, 37].

#### 3.4.6. Wetting Time

Wetting time, a crucial indicator of the tablet's disintegration ease, reflects the time taken for a tablet to fragment when placed on the tongue without movement. The recorded wetting time values for escitalopram oxalate tablets ranged from 27 s (ODT3) to 58.6 s (ODT9), as presented in Table 4. Wetting time indicates the hydrophilicity of the tablet as the wetting time decreases; then, the formula becomes more hydrophilic. Furthermore, the wetting time is longer for the formula that has higher hardness such as ODT9 [30].

#### 3.4.7. Water Absorption Ratio

The water absorption ratio values for the escitalopram oxalate tablets fell within the range of 55.16% for ODT4 to 153.46% for ODT9, as indicated in Table 4. It is noteworthy that these results align with the established criteria for ODTs. The water absorption ratio is a crucial parameter reflecting the tablets' ability to absorb water, a characteristic desirable for effective disintegration [36]. Also, water absorption ability almost correlates with wetting ability as the formula has a higher wetting rate that is more hydrophilic, and it is expected to absorb more water. That is represented by ODT9 which has a faster disintegration rate, and it is considered the optimum formula to prepare an ODT [30].

#### 3.4.8. Drug Content

The results of drug content for the prepared formulations were set between 98.04% and 99.59% as illustrated in Table 4. These values were within the acceptable range outlined in the USP which stated that the drug content should be within 85%–115% [36].

The values of escitalopram oxalate tablets based on drug content ranged from 98.04 for ODT1 to 99.59 for ODT5 and all these values were in the acceptable range.

#### 3.4.9. In Vitro Release of Escitalopram Oxalate

In vitro release examinations of escitalopram ODTs were performed at 37°C by the USP Dissolution Tester, Apparatus II (paddle), and operating 900 mL of phosphate buffer (pH 7.4) as the dissolution medium and at a rotation rate of 50 rpm. The release of escitalopram oxalate tablets from the various formulations was judged by determining the amount of drug released as a percentage.

**Figure 2:**
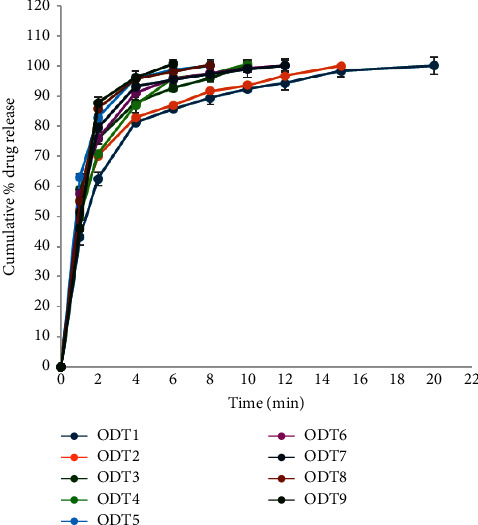
In vitro drug release from the nine prepared escitalopram oxalate tablet formulas.

The amount of drug release was determined utilizing the equation generated from the standard calibration curve. The observations for different formulas are shown in Figure 2.

Furthermore, Table 5 illustrates T50, T80, and T100 for the nine prepared formulas, representing the time required until 50%, 80%, and 100% of escitalopram oxalate were released from each formula, respectively. As shown in the table, generally by increasing the concentration of the superdisintegrant, the time required for the drug to be released from the formula was decreased. Specifically, T80 for escitalopram oxalate tablet formulations (ODT1, ODT4, and ODT7) containing a 2.5% superdisintegrant demonstrated time of 3.86, 3.13, and 2.08 min, respectively. The increment of superdisintegrant concentration from 2.5% to 5% in formulations (ODT2, ODT5, and ODT8) resulted in enhanced drug release, which was indicated by a lower time for T80, 3.56, 2.85, and 1.81 min for the mentioned formulations. Further escalation of the concentration of superdisintegrant reaching 7.5% in escitalopram oxalate formulations (ODT9, ODT6, and ODT3) led to T80 values of 2.69, 2.53, and 1.81, respectively.

Observations revealed a trend of rapid drug release with increasing superdisintegrant concentration from 2.5% to 7.5%, except in formulations with crospovidone, where drug release increased up to 5% concentration, and further increments led to a decrease in the release. These findings corroborate the in vitro disintegration test results, affirming that the ninth formula which consisted of 7.5% sodium starch glycolate, emerged as the optimal formulation.

Fast release of the active agent was markedly noted in the preparations containing sodium starch glycolate, then the one with crospovidone and finally the formula with croscarmellose sodium. Croscarmellose sodium and sodium starch glycolate rely heavily on swelling for their disintegration action, while crospovidone focuses more on wicking and rapid water uptake without significant swelling.

Crospovidone generally offers the fastest disintegration due to its mechanism, which does not involve gel formation.

ODT9 exhibited the ultimate dissolution rate (100%) after 6 minutes. This fast dissolution rate of the drug is mainly due to the simple fragmentation of the tablets and swift drug absorption by the dissolution medium. Across all formulated ODTs, the release of the drug reached approximately 90%–100% after 10 minutes.

According to the previous examinations, ODT9 emerged as the superior formulation among the tested escitalopram oxalate tablets. This formulation exhibited optimal micrometric characteristics, superior results in quality control parameters for escitalopram oxalate, and the best in vitro release profile of the active agent.

In the above ODT formulations, sodium starch glycolate (Primojel) may have facilitated the fastest drug release due to its rapid and substantial swelling action, which led to immediate tablet disintegration and increased the surface area available for drug dissolution. While crospovidone (Polyplasdone XL) is also fast due to its wicking action, it may not swell as much, resulting in slightly slower drug release. Croscarmellose sodium (Ac-Di-Sol), which relies on both swelling and wicking, might have been the slowest because its swelling action is not as immediate or pronounced as that of sodium starch glycolate [14, 38].

#### 3.4.10. Kinetic Treatments and Parameters for the In Vitro Release of Escitalopram Oxalate ODTs

The determination of the optimal kinetic order for the in vitro release profile of escitalopram oxalate formulations is generally inferred from the greatest values of the calculated correlation coefficients. The results indicated that all prepared formulations of escitalopram oxalate ODT follow Higuchi's diffusion model. This is mainly due to quick diffusion through the porous structure of the tablets. The highest magnitude of correlation coefficients of the prepared escitalopram oxalate tablet formulas was ODT1 (0.978259), ODT2 (0.984225), ODT3 (0.986421), ODT4 (0.989704), ODT5 (0.971331), ODT6 (0.965907), ODT7 (0.950578), ODT8 (0.962796), and ODT9 (0.978859), respectively.

#### 3.4.11. Accelerated Stability Studies of Escitalopram Oxalate ODTs

For accelerated stability studies, ODT9, which showed the superior properties of all the nine prepared escitalopram oxalate tablet formulas, was placed in Petri dishes and kept in two ovens adjusted at 40°C and 60°C. Moreover, the humidity was controlled to 75% by utilizing a saturated solution of sodium chloride [39]. Samples from each formula were withdrawn at regular periods of 1, 2, 3, 4, 5, and 6 months. The samples were tested in terms of hardness, in vitro disintegration time, in vitro release, and drug content.

The hardness of escitalopram oxalate formulas at the two controlled temperatures (40°C and 60°C) is explained in Table 6. It was found that the hardness after 6 months at the two mentioned temperatures was larger than those measured at zero time. It was also found that the hardness of formulas kept at 60°C was higher than that of those kept at 40°C. That correlated with the literature as the hardness was increased over time and by increasing the storage temperature.

The in vitro disintegration time of the escitalopram oxalate formula at the two raised temperatures (40°C and 60°C) is shown in Table 6. The results indicated that the in vitro disintegration time after 6 months at the two elevated temperatures was larger than those measured at zero time. The results also showed that the time of in vitro disintegration of formulas kept at 60°C was higher than that of those kept at 40°C. The in vitro disintegration times of the escitalopram oxalate formula calculated after the different periods were affected by the calculated hardness for the same escitalopram oxalate formula. That is, increasing the hardness by storage will also increase the time of the in vitro disintegration.

T50 and T80 in minutes of the in vitro drug release for ODT9 at the two raised temperatures (40°C and 60°C) are shown in Table 7. It was found that there was a minor decrease in the release of escitalopram oxalate after storage at these two elevated temperatures for 6 months. It was also found that the drug release of escitalopram oxalate formula kept at 60°C was lower than those kept at 40°C.

**Figure 3:**
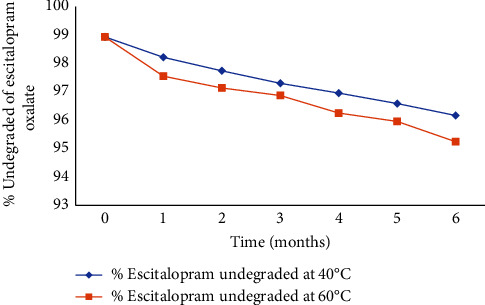
Percent escitalopram undegraded after accelerated stability testing for 6 months for ODT9.

Figure 3 illustrates the percent undegraded escitalopram oxalate in ODT9 at the raised temperatures (40°C and 60°C).

The kinetic treatment for the degradation of escitalopram oxalate for ODT9 at both 40°C and 60°C was shown in different kinetic orders: zero, first, and second orders. Data in Table 8 illustrated the kinetic treatments, while Table 9 illustrated the kinetic parameters for the stability study of prepared escitalopram oxalate tablet ODT9. The kinetic treatment involved plotting time in months against the degraded percent of escitalopram oxalate in terms of zero order, time against log percent of escitalopram undegraded regarding the first order, and time against the reciprocal of the percent of escitalopram undegraded in case of the second order. The analysis revealed that all escitalopram oxalate formulations conformed to zero-order kinetics in the stability study, as determined by the greatest values of the calculated correlation coefficients, as shown in Table 8. This suggests that the degradation of escitalopram oxalate in these formulations is primarily independent of the drug concentration (zero order).

The zero-order reaction is related to the rate which is constant and independent of the undegraded drug remaining. *K* was calculated at each temperature. *K*_20_ was calculated using the Arrhenius equation [32]:

(8) $$\begin{array}{c} \log\;K=\log\;A-\left[\left(\frac{\text{Ea}}{2.303}\right)\left(\frac{1}{RT}\right)\right],\\ \log\left(\frac{K_{2}}{K_{1}}\right)=\frac{\left(\text{Ea}/2.303R\right)}{\left[\left(T_{2}-T_{1}\right)/\left(T_{2}.T_{1}\right)\right]}, \end{array}$$

where *K* is the stability constant, *A* is the Arrhenius factor, Ea is the energy of activation, *R* is the gas constant, and *T* is the absolute temperature (*T*_1_ refers to 40°C, and *T*_2_ refers to 60°C). Furthermore, the time of half-life and shelf-life were estimated by employing the subsequent relations and shown in Table 9:

(9) $$\begin{array}{c} t_{1/2}=\frac{a}{2k},\\ t_{90}=\frac{a}{10K}, \end{array}$$

where (*a*) is the initial drug amount of drug.

## 4. Conclusion

The ODT emerges as a promising drug delivery system, facilitating direct drug release in the oral cavity and offering numerous advantages, particularly in improving the rate of dissolution and bioavailability of hydrophobic medications. This delivery method holds significant appeal for specific patient populations, particularly those with difficulties swallowing traditional tablets.

In our investigation, we focused on the development of ODTs for the hydrophobic drug, escitalopram oxalate, employing a strategy centered on superdisintegrants. Utilizing the direct compression method, we prepared oral tablets of escitalopram oxalate and conducted a comprehensive analysis of the formulations.

The micrometric characterization of the powder blend used in the ODT formulations was carefully examined, followed by a detailed assessment of quality control investigations.

This study underscores the potential of tailored ODT formulations, specifically emphasizing the crucial role of superdisintegrants in enhancing the overall performance of hydrophobic drugs and fabricating them in suitable delivery systems.

However, more investigations should be performed soon to confirm the efficiency of this ODT formula. For example, in vivo pharmacokinetic and pharmacodynamic studies should be obtained to evaluate the bioavailability, absorption rate, and onset of action of the ODT formulation concerning conventional tablets. Furthermore, an assessment of the clinical effectiveness and safety of the ODT formulation in patients with anxiety or depressive disorders is recommended. Large-scale manufacturing optimization and evaluation of patient compliance and acceptance are also useful to be obtained.

## Figures and Tables

**Table 1:** The nine prepared formulas of escitalopram oxalate ODTs.

Ingredient (mg/tablet)	ODT1	ODT2	ODT3	ODT4	ODT5	ODT6	ODT7	ODT8	ODT9
Escitalopram oxalate	15								
Croscarmellose sodium	5	10	15	—	—	—	—	—	—
Crospovidone	—	—	—	5	10	15	—	—	—
Sodium starch glycolate	—	—	—	—	—	—	5	10	15
Avicel pH 102	25								
Aspartame	2								
Talc	4								
Magnesium stearate	2								
Mannitol	67	62	57	67	62	57	67	62	57
Total	120	120	120	120	120	120	120	120	120

**Table 2:** Effect of Carr's index, Hausner's ratio, and angle of repose on flow character.

Carr's index (%)	Flow character	Hausner's ratio	Flow character	Angle of repose	Flow character
< 10	Excellent	1.00–1.11	Excellent	<25°	Excellent
11–15	Good	1.12–1.18	Good	25°–30°	Good
16–20	Fair	1.19–1.25	Fair	30°–40°	Passable
21–25	Passable	1.26–1.34	Passable	> 40°	Very poor
26–31	Poor	1.35–1.45	Poor		
32–37	Very poor	1.46–1.59	Very poor		
> 38	Very very poor	> 1.600	Very very poor		

**Table 3:** The data collected for the angle of repose, the bulk and the tapped densities, Hausner ratio, and Carr's index of the escitalopram oxalate powder blend formulas.

Formulation	Angle of repose (*θ*)	Bulk density (gm/cm^3^)	Tapped density (gm/cm^3^)	Hausner ratio (HR)	Carr's index (CI)
ODT1	34.18° ± 0.26	0.486 ± 0.004	0.591 ± 0.003	1.231	17.095
ODT2	37.52° ± 0.35	0.462 ± 0.005	0.585 ± 0.007	1.228	16.925
ODT3	39.75° ± 0.58	0.510 ± 0.004	0.635 ± 0.005	1.240	21.000
ODT4	35.51° ± 0.28	0.427 ± 0.007	0.578 ± 0.003	1.374	27.667
ODT5	38.42° ± 0.62	0.445 ± 0.008	0.552 ± 0.004	1.293	23.609
ODT6	39.21° ± 0.85	0.465 ± 0.008	0.612 ± 0.012	1.335	25.545
ODT7	38.19° ± 0.34	0.427 ± 0.004	0.572 ± 0.008	1.358	26.833
ODT8	35.12° ± 0.28	0.473 ± 0.003	0.585 ± 0.006	1.251	18.444
ODT9	32.96° ± 0.18	0.482 ± 0.004	0.566 ± 0.003	1.168	16.094

**Table 4:** The postcompression parameters of the nine prepared escitalopram oxalate ODTs.

Parameter	ODT1	ODT2	ODT3	ODT4	ODT5	ODT6	ODT7	ODT8	ODT9
Weight variation (mg)	199.57 ± 2.16	200.92 ± 1.87	201.09 ± 1.66	200.77 ± 1.76	200.12 ± 1.854	200.38 ± 1.76	202.07 ± 1.97	200.88 ± 1.61	201.82 ± 2.49
Thickness (mm)	3.84 ± 0.012	3.82 ± 0.011	3.83 ± 0.01	3.88 ± 0.01	3.85 ± 0.01	3.85 ± 0.01	3.84 ± 0.01	3.81 ± 0.01	3.82 ± 0.01
Diameter (mm)	8.07 ± 0.01	8.08 ± 0.01	8.08 ± 0.01	8.08 ± 0.01	8.08 ± 10.01	8.08 ± 0.01	8.07 ± 0.01	8.07 ± 0.01	8.08 ± 0.01
Hardness (kg/cm^2^)	4.25 ± 0.19	4.51 ± 0.20	4.40 ± 0.19	4.37 ± 0.18	4.21 ± 0.10	4.55 ± 0.24	4.55 ± 0.21	4.48 ± 0.22	4.34 ± 0.24
Friability (%)	0.79	0.41	0.51	0.53	0.41	0.37	0.68	0.41	0.37
Disintegration time (sec.)	21.90 ± 0.88	18.00 ± 0.94	15.60 ± 0.70	13.30 ± 0.82	10.50 ± 0.71	11.80 ± 0.79	9.80 ± 0.63	8.90 ± 0.74	8.30 ± 0.82
Wetting time (sec.)	36.40 ± 0.89	32.80 ± 0.84	27.00 ± 0.71	45.40 ± 0.55	34.60 ± 0.55	43.00 ± 2.00	34.00 ± 0.71	42.6 ± 01.82	58.60 ± 1.14
Water absorption ratio (%)	60.80 ± 0.53	78.12 ± 3.12	90.03 ± 1.16	55.16 ± 2.74	64.82 ± 0.91	78.30 ± 3.14	88.80 ± 3.23	130.98 ± 3.39	153.46 ± 2.42
Drug content (%)	98.04 ± 0.89	99.02 ± 0.58	99.42 ± 0.67	98.87 ± 0.49	99.59 ± 0.47	98.72 ± 1.07	99.10 ± 0.23	98.99 ± 0.89	98.88 ± 0.94

**Table 5:** T50, T80, and T100 in minutes of the in vitro drug release for the nine prepared escitalopram oxalate tablet formulas.

Formula	ODT1	ODT2	ODT3	ODT4	ODT5	ODT6	ODT7	ODT8	ODT9
T50	1.35	0.96	0.85	1.00	0.79	0.87	0.98	0.91	1.09
T80	3.86	3.56	2.69	3.13	2.85	2.53	2.08	1.81	1.81
T100	19.76	14.97	9.86	9.62	7.78	11.83	11.70	7.75	7.69

**Table 6:** Stability study of the hardness and in vitro disintegration time of escitalopram oxalate tablets ODT9 at 40°C and 60°C.

	Initial	1 month	2 months	3 months	4 months	5 months	6 months
Hardness (kg/cm^2^)							
40°C	4.37 ± 0.04	4.49 ± 0.03	4.53 ± 0.02	4.71 ± 0.11	4.85 ± 0.09	5.28 ± 0.02	5.41 ± 0.01
60°C	4.37 ± 0.04	4.56 ± 0.01	4.53 ± 0.16	4.695 ± 0.01	5.015 ± 0.37	5.335 ± 0.01	5.54 ± 0.10
In vitro disintegration time (seconds)							
40°C	8.6 ± 0.42	9.35 ± 0.21	10.9 ± 0.57	14.6 ± 1.56	16.5 ± 1.31	18.6 ± 1.56	20.8 ± 0.14
60°C	8.6 ± 0.42	10.95 ± 0.50	12.5 ± 0.57	15.5 ± 1.13	18.4 ± 1.56	22.3 ± 0.71	26.5 ± 1.31

**Table 7:** T50 and T80 in minutes of the stability study for the in vitro drug release of escitalopram oxalate from ODT9 at 40 and 60.

Time	0 (fresh)	1 month	2 months	3 months	4 months	5 months	6 months
40°C							
T50	0.96	0.85	1.00	0.79	0.87	0.98	0.91
T80	3.56	2.69	3.13	2.85	2.53	2.08	1.81
60°C							
T50	0.99	1.01	1.06	1.11	1.18	1.25	1.31
T80	1.81	1.86	1.93	1.99	2.32	2.82	3.67

**Table 8:** The calculated correlation coefficients for the degradation of escitalopram oxalate from ODT9 at 40°C and 60°C employing different kinetic orders.

Correlation coefficients (*r*)					
Zero-order		First-order		Second-order	
40°C	60°C	40°C	60°C	40°C	60°C
0.997987	0.988121	−0.99764	−0.987744	0.997824	0.987358

**Table 9:** Data calculating the shelf-lives of escitalopram oxalate tablets for ODT9.

*K*_40_ month^−1^	*K*_60_ month^−1^	Ea cal/mole	*k*_20_ month^−1^	*t*_1/2_ month	*t*_90_ month
0.415143	0.435714	500.8967	0.39294	127.2458	25.44916

## Data Availability

The data that support the findings of this study are available on request from the corresponding author. The data are not publicly available due to privacy or ethical restrictions.
